# Complete genome analysis of African swine fever virus genotypes II, IX and XV from domestic pigs in Tanzania

**DOI:** 10.1038/s41598-023-32625-1

**Published:** 2023-03-31

**Authors:** Jean N. Hakizimana, Clara Yona, Mariam R. Makange, Ester A. Kasisi, Christopher L. Netherton, Hans Nauwynck, Gerald Misinzo

**Affiliations:** 1 OR Tambo Africa Research Chair for Viral Epidemics, SACIDS Foundation for One Health, Sokoine University of Agriculture, PO Box 3297, Morogoro, Tanzania; 2 Department of Biosciences, Solomon Mahlangu College of Natural and Applied Sciences, Sokoine University of Agriculture, PO Box 3038, Morogoro, Tanzania; 3 SACIDS Africa Centre of Excellence for Infectious Diseases, SACIDS Foundation for One Health, Sokoine University of Agriculture, PO Box 3297, Morogoro, Tanzania; 4 African Swine Fever Vaccinology Group, The Pirbright Institute, Ash Road, Pirbright, Woking, GU24 0NF Surrey UK; 5 Laboratory of Virology, Faculty of Veterinary Medicine, Ghent University, Salisburylaan 133, 9820 Merelbeke, Belgium; 6 Department of Veterinary Microbiology, Parasitology and Biotechnology, College of Veterinary Medicine and Biomedical Sciences, Sokoine University of Agriculture, PO Box 3019, Morogoro, Tanzania

**Keywords:** Molecular biology, Evolution, Phylogenetics

## Abstract

African swine fever (ASF) caused by ASF virus (ASFV) is an infectious transboundary animal disease notifiable to the World Organization for Animal Health causing high mortality in domestic pigs and wild boars threatening the global domestic pig industry. To date, twenty-four ASFV genotypes have been described and currently genotypes II, IX, X, XV and XVI are known to be circulating in Tanzania. Despite the endemic status of ASF in Tanzania, only one complete genome of ASFV from the country has been described. This study describes the first complete genome sequence of ASFV genotype XV. In addition, the first Tanzanian complete genome of ASFV genotype IX and three ASFV strains belonging to genotype II collected during ASF outbreaks in domestic pigs in Tanzania were determined in this study using Illumina sequencing and comparative genomics analysis. The generated ASFV complete genome sequences ranged from 171,004 to 184,521 base pairs in length with an average GC content of 38.53% and encoded 152 to 187 open reading frames. The results of this study provide insights into the genomic structure of ASFV and can be used to monitor changes within the ASFV genome and improve our understanding of ASF transmission dynamics.

## Introduction

The global domestic pig industry is threatened by African swine fever (ASF), an infectious transboundary animal disease notifiable to the World Organization for Animal Health (WOAH) representing a serious threat to domestic pigs industry and wild boars^1,2^. The soft ticks of the *Ornithodoros moubata* complex and African wild suids including warthogs (*Phacochoerus* spp.), bush pigs (*Potamochoerus* spp.) and giant forest hogs (*Hylochoerus meinertzhageni*) have been reported to be infected by ASF virus (ASFV), the etiological agent of ASF without causing disease^3,4^. Currently, ASFV is the only known DNA arbovirus and the only member of the family of *Asfarviridae*, genus *Asfivirus*^5^. Since the description of the first case of ASF in Kenya in 1921, it has been reported by more than 33 countries of Africa, South of the Sahara with two epidemics outside the African continent^4^. The first wave of ASF spread outside Africa was reported in 1957 and 1960 in Portugal with subsequent spread to neighboring European countries and South America^6,7^. Interestingly, ASF was eradicated in those countries except in Sardinia Island, Italy. In 2007, a new wave of ASF started in Georgia and subsequently spread to other European and Asian countries with devastating economic impact on the global domestic pig industry^8–10^. In February 2021, ASF was reported in wild bearded pigs (*Sus barbatus*) on the island of Borneo, South-Eastern Asia^11^. In April 2021, ASF was reported in the Dominican Republic and reached Haiti in August 2021 threatening neighbouring South American countries and mainland North America^12^. To date, twenty four ASFV genotypes have been described and genotypes I and II are known to be circulating outside the African continent^8,13^. The first transcontinental spread of ASFV in 1957 and 1960 was due to genotype I while on the other hand, the ASFV introduced into Georgia in 2007 was identified as genotype II^14^. ASFV genotypes II was introduced into China in 2018 with devastating socioeconomic impact, and since then genotype I isolates have also been reported^15,16^. In Italy, the ASFV genotype I remained endemic in the Sardinia Island since 1978 and ASFV genotype II was identified for the first time in mainland Italy in 2022^17,18^.

ASFV genotype I has been reported to be predominant in western and central Africa and recently ASFV genotype II has been reported for the first time in Nigeria^19^. ASFV genotype IX has been reported in central and eastern African countries including in the Republic of Congo (Brazzaville) and the Democratic Republic of the Congo^20^. In eastern and southern Africa, a sylvatic cycle between wild suids and soft ticks of the *Ornithodoros moubata* complex plays an important role as an ASFV reservoir^4^. The cohabitation between warthogs and soft ticks in wildlife protected areas in eastern and southern Africa represents a permanent risk for ASFV transmission from sylvatic hosts to the domestic pig population^4,21^. Despite the existence of the ASFV sylvatic cycle in eastern and southern Africa, the predominance of the ASFV domestic cycle in most of the reported ASF outbreaks has been documented in several countries including Tanzania^22–24^. In Tanzania, ASFV genotypes II, IX, X, XV and XVI have been reported in different epidemiological situations based on partial genome sequencing and only one isolate of genotype II from the country has been subjected to complete genome sequencing^22,24–29^. Complete genome sequences of ASFV are critical for understanding the viral genomic structure and transmission dynamics in order to monitor changes within the ASFV genome and improve ASF risk management strategy. The objective of this study was to analyze the complete genome sequences of ASFV genotypes II, IX and XV from domestic pigs in Tanzania.

## Results

### Complete genome sequences of the Tanzanian ASFV strains from ASF outbreaks in domestic pigs

In this study, we report the complete genome sequences of five ASFV isolates obtained from domestic pigs from Tanzania that were collected between 2008 and 2020. The ASFV isolate Tan/08/Mazimbu was obtained during the 2008 ASF outbreak in domestic pigs in Morogoro region, Tanzania and was classified into ASFV genotype XV using partial nucleotide amplification and sequencing targeting the *B646L* (p72) gene as previously reported^30^. In addition, the complete genome sequences of ASFV genotypes II (TAN/17/Kibaha, TAN/17/Mbagala and TAN/20/Morogoro), and IX (TAN/16/Magu) are reported in this study. The generated ASFV complete genomes ranged from 171,004 to 184,521 base pairs (bp) in length with an average GC content of 38.53% and genome coverage from 33 to 108 times (Table 1). The assembled genomes belonging to ASFV genotype II had a length of 184,521 bp for TAN/17/Kibaha, 171,004 bp for TAN/17/Mbagala and 183,389 bp for TAN/20/Morogoro with a GC content of 38.46, 38.88 and 38.54%, respectively and contained 166 to 187 open reading frames (ORFs). The Tanzanian ASFV genotype IX TAN/16/Magu strain was 182,514 bp long with 38.65% GC content and 157 ORFs, while the genotype XV Tan/08/Mazimbu strain was 178,832 bp long with 38.15% GC content and 152 ORFs. Sequences generated in this study have been submitted to NCBI GenBank and assigned accession numbers as shown in Table 1.

**Table 1:** Summary of Tanzanian ASFV complete genomes characterized in this study.

Strain name	Total number of reads	Number of ASFV specific reads	Mean bases phred quality score	ASFV Genome size (bp)	GC content (%)	Number of ORFs	GenBank accession no	Mean genome coverage	P72 genotype
TAN/17/Kibaha	19,760,822	57,260	35.9	184,521	38.46	187	ON409979	46	II
TAN/17/Mbagala	20,375,542	37,932	35.8	171,004	38.88	166	ON409982	33	II
TAN/20/Morogoro	28,139,542	42,428	36.4	183,389	38.54	186	ON409983	36	II
TAN/16/Magu	16,774,780	113,960	35.9	182,514	38.65	157	ON409980	90	IX
TAN/08/Mazimbu	69,217,300	160,185	36	178,830	38.15	152	ON409981	108	XV

Within each genome of TAN/17/Kibaha and TAN/20/Morogoro strains, a total of 44 members of different multigene families (MGFs) were identified including 3 members of MGF 100, 12 members of MGF 110, 3 members of MGF 300, 18 members of MGF 360 and 10 members of MGF 505. In the genome of the strain TAN/17/Mbagala characterized in this study, a total of 34 MGFs were identified including 2 members of the MGF 100, 3 members of MGF 300, 19 members of MGF 360 and 10 members of MGF 505. The TAN/16/Magu strain had a total of 40 MGFs including 3 members of MGF 100, 11 members of MGF 110, 3 members of MGF 300, 14 members of MGF 360 and 9 members of MGF 505. Furthermore, the Tan/08/Mazimbu strain had a total of 37 MGFs including 2 members of MGF 100, 10 members of MGF 110, 2 members of MGF 300, 16 members of MGF 360 and 7 members of MGF 505.

### Comparative viral genomics analysis

TAN/17/Kibaha exhibited 99.95% nucleotide identity and 100% query coverage with the ASFV genotype II reference genome Georgia 2007/1 (GenBank accession number FR682468.2) with more than 99.90% identity to genotype II ASFV isolates from Tanzania, Malawi, and several European and Asian countries. Analysis of the ASFV strains TAN/20/Morogoro and TAN/17/Mbagala revealed more than 99.90% nucleotide identity with several ASFV genotype II circulating in Africa, Europe and Asia. The Tanzanian ASFV genotype IX TAN/16/Magu strain was closely related to isolates responsible for the 2015 ASF outbreak in Uganda including ASFV R35 isolate (GenBank accession number MH025920.1) with 99.85% nucleotide identity and 100% query coverage and the isolate Ken06.Bus (GenBank accession number NC_044946.1) collected in 2006 during ASF outbreak in Kenya with 99.90% nucleotide identity. Furthermore, the ASFV isolate most closely related to Tan/08/Mazimbu strain at the NCBI GenBank was MalawiLil-20/1(1983) (GenBank accession number AY261361) with 96.93% nucleotide identity and 99% query coverage.

**Figure 1:**
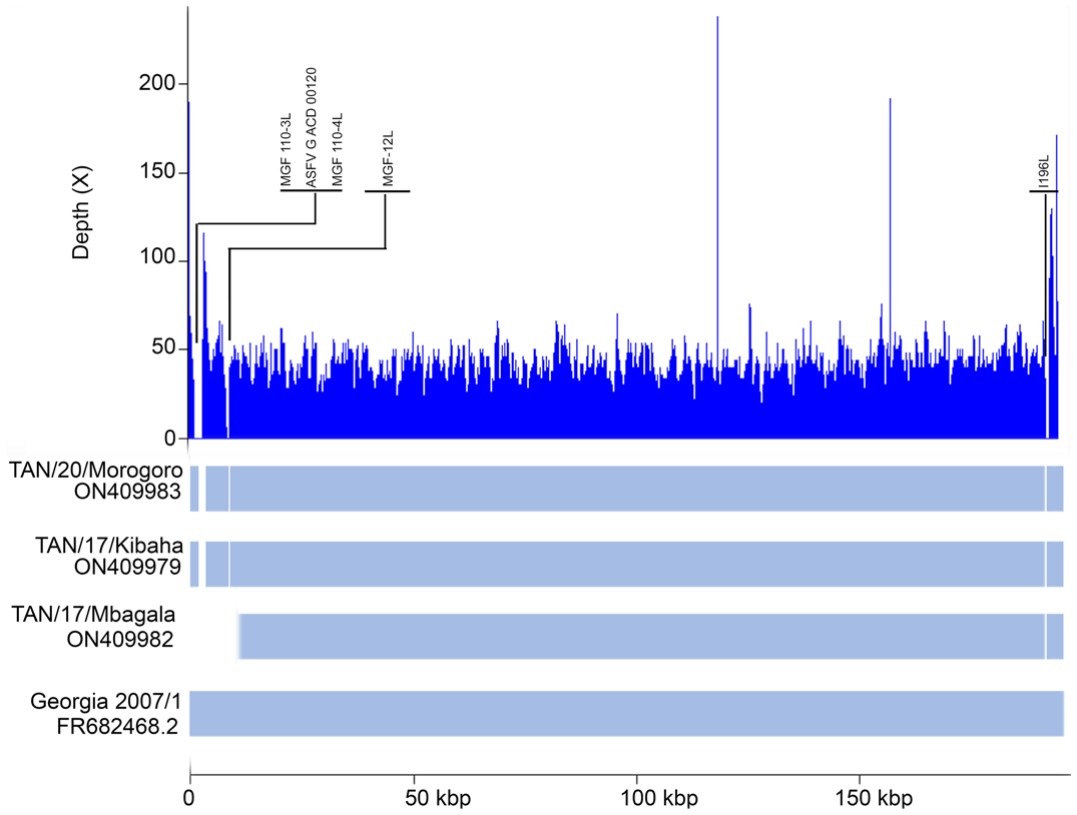
Coverage depth representation of the TAN/20/Morogoro ASFV strain along with other ASFV strains belonging to genotype II described in this study. The coverage was calculated using samtools version 1.10 and plotted using the lattice package in RStudio.

With a genome size of 184,521 bp, the strain TAN/17/Kibaha is 6063 bp shorter than the ASFV genotype II reference genome Georgia 2007/1 (190,584 bp). The difference in genome size is due to the overhang at the 5′ (3357 bp) and 3′ (1894 bp) ends present in the reference genome and could not be sequenced in the TAN/17/Kibaha strain. The overhang ends that were not sequenced were 3983 and 2523 bp long at 5′ and 3′ ends, respectively for the TAN/20/Morogoro strains, while 17,641 bp at the 5′ end and 1828 bp at the 3′ end could not be sequenced for the strain TAN/17/Mbagala. In addition, other insertions and deletions (indels) were observed throughout the alignment of the reference genome Georgia 2007/1 and genotype II strains described in this study including deletion of a 686 bp fragment between the positions 8346 and 9030 (Fig. 1). This deletion has led to the truncation of three genes (*MGF 110 3L-4L* and *ASFV G ACD 00120*). A similar large deletion was reported in the Tanzania/Rukwa/2017/1 isolate (GenBank accession number LR813622) responsible for the 2017 ASF outbreak in domestic pigs in South-western Tanzania. At position 6983, an insertion of 2 bp (TG) in the TAN/20/Morogoro strains was observed while these bases are similarly absent in TAN/17/Kibaha and Georgia 2007/1. At position 9646, a 3 bp (GAT) deletion was observed in TAN/17/Kibaha and TAN/20/Morogoro. Small fragments of 5 (CCCCC) and 3 (CCC) bp were deleted in TAN/17/Mbagala and TAN/20/Morogoro, respectively at position 14,253. A similar insertion of one base (A) at position 15,174 in the TAN/17/Kibaha and TAN/20/Morogoro genome sequences was observed. A deletion of 7 bp (CCCCCCC) in TAN/17/Kibaha and TAN/20/Morogoro between the positions 15,695 and 15,701 was observed and 2 bp (GG) absent in the reference genome were inserted at the site 17,651 in the TAN/20/Morogoro strain while at the same position, 1 bp was deleted in the strain TAN/17/Kibaha (Supplementary Table 1). At position 17,870, 2 bp (GG) absent in other genomes were inserted in the strain TAN/20/Morogoro. A similar deletion of 65 bp was detected in all strains characterized in this study between the positions 176,450 and 176,515 compared to the reference genome Georgia 2007/1 (Fig. 1).

**Figure 2:**
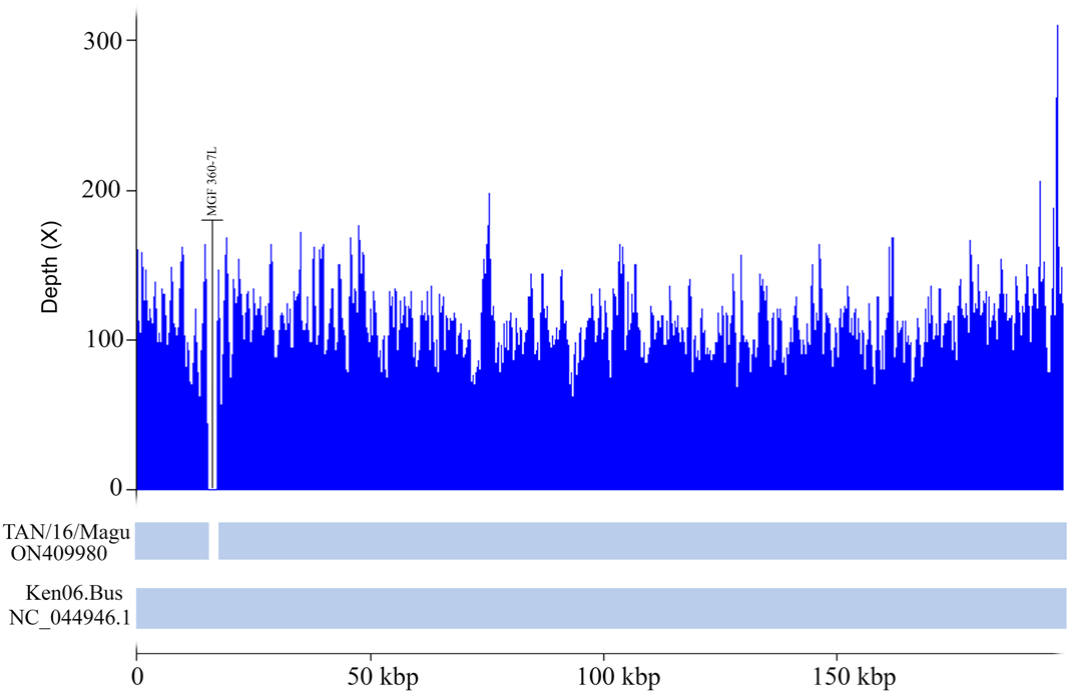
Coverage depth representation of the ASFV strain of genotype IX described in this study (TAN/16/Magu). The coverage was calculated using samtools version 1.10 and plotted using the lattice package in RStudio.

The complete genome sequence of the Tanzanian ASFV genotype IX TAN/16/Magu was compared to the Ugandan ASFV isolate R35 (GenBank accession number MH025920.1) and Ken06.Bus (GenBank accession number NC_044946.1). TAN/16/Magu is 6115 bp shorter than R35 mainly due to overhang at 5′ (1944 bp) and 3′ (1983 bp) ends that could not be sequenced for the strain TAN/16/Magu. In addition, the strain TAN/16/Magu lacks a large fragment of 1969 bp present in R35 and Ken06.Bus isolates between the alignment positions 14,134 and 16,103 (Supplementary Table 1). The deleted fragment covers MGF 360-7L (Fig. 2). Between positions 41,277 and 41,340, a deletion of a 63 bp fragment was detected in TAN/16/Magu as compared to R35. A similar fragment is absent in pig-derived genotype IX Kenyan ASFV isolate Ken06.Bus.

**Figure 3:**
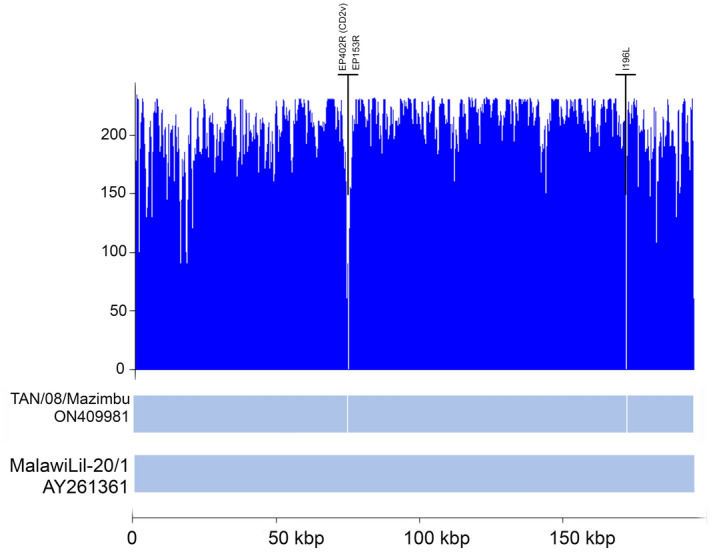
Coverage depth representation of the ASFV strain of genotype XV described in this study (Tan/08/Mazimbu). The coverage was calculated using samtools version 1.10 and plotted using the lattice package in RStudio.

With a genome size of 178,830 bp, the strain Tan/08/Mazimbu is within the range of previously reported length of ASFV complete genome sequences (between 170 and 194 kilobase pairs). Compared to the most closely related ASFV isolate MalawiLil-20/1(1983), the strain Tan/08/Mazimbu is 8780 bp shorter with several indels and single nucleotide polymorphisms (SNP) along the alignment. Overhangs that could not be sequenced for Tan/08/Mazimbu were 417 and 354 bp at the 5′ and 3′ ends, respectively. Most of the variations were located at the left and right termini including the deletion of 326 bp between the alignment positions 71,360 and 71,686 and 177 bp between the alignment positions 174,711 and 174,888 (Fig. 3). Sanger sequencing was used to exclude the gap through the *EP402R (CD2v)* gene using a specific primers pair.

### Phylogenetic analysis

**Figure 4:**
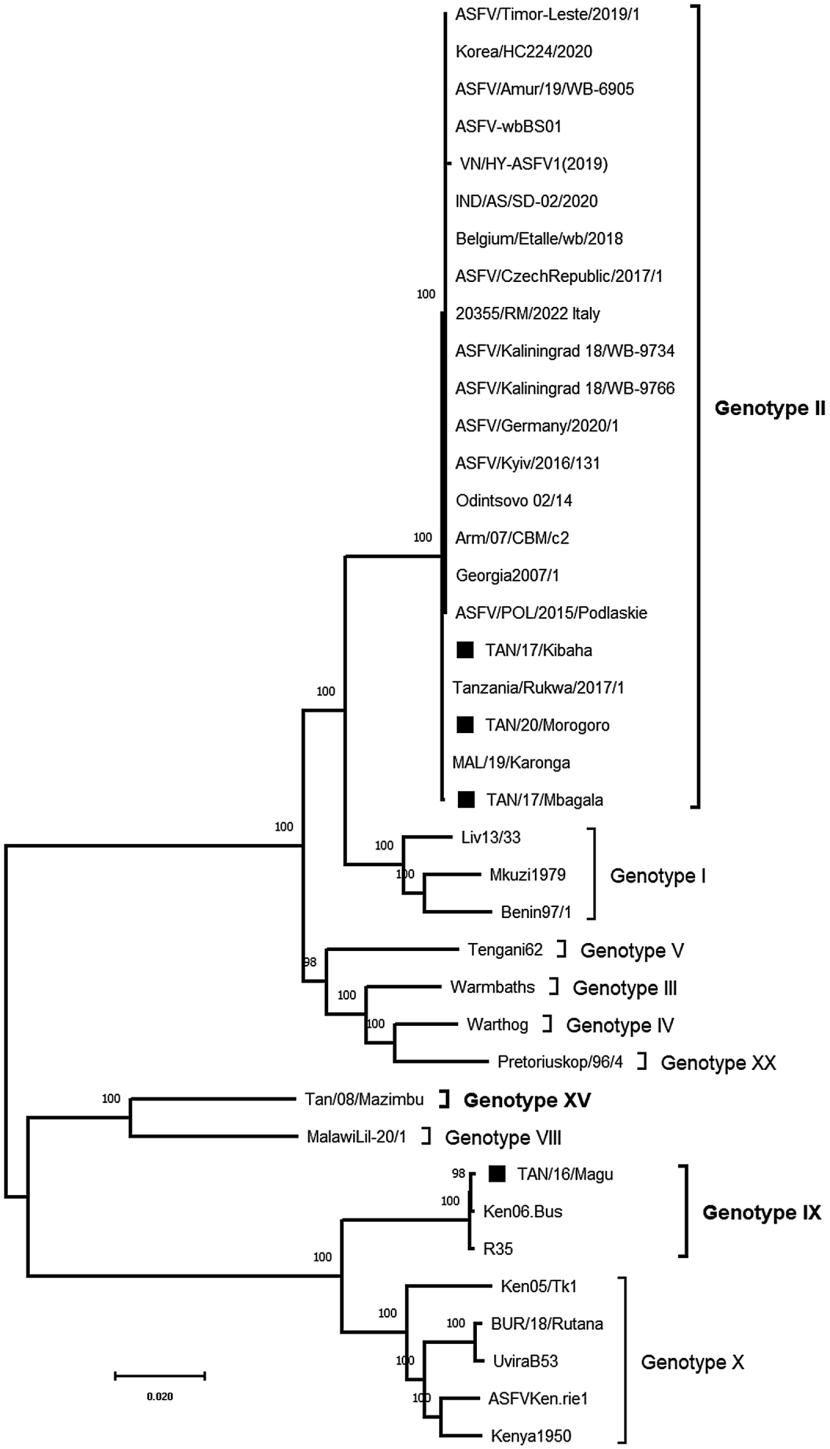
Maximum likelihood phylogenetic tree obtained using selected ASFV complete genome sequences representing each ASFV genotype with available complete genomes. Sequences generated in this study are represented by black squares and the scale bar indicates nucleotide substitution per site while the node values show the percentage of bootstrap support.

The Tanzanian ASFV strains described in this study were classified along with previously reported ASFV genotypes using complete genome sequences (Fig. 4). The strains TAN/17/Kibaha, TAN/17/Mbagala and TAN/20/Morogoro clustered into ASFV genotype II, while TAN/16/Magu and Tan/08/Mazimbu clustered into genotypes IX and XV, respectively. The full genome phylogeny of isolates characterized in this study matches that obtained by partial nucleotide amplification and sequencing targeting the *B646L* (p72) gene as previously reported^24,30^. In addition, the ASFV isolates characterized in this study clustered within ASFV primary clade I for the strain TAN/16/Magu, primary clade II subclade 2 for the strains TAN/17/Kibaha, TAN/17/Mbagala and TAN/20/Morogoro while the strain Tan/08/Mazimbu clustered with the isolate MalawiLil-20/1(1983) which was previously classified as a singleton^31^.

The *K145R* and *O174L* genes were selected for further discrimination among the ASFV genotype II isolates as previously described^32–34^. Analysis of the *K145R* genes of all ASFV genotype II isolates characterized in this study showed 100% nucleotide identity to the reference genome Georgia 2007/1 belonging to *K145R* variant I. On the other hand, the *O174L* gene analysis revealed that all ASFV genotype II described in this study were 100% homologous to the reference genome Georgia 2007/1 representing the variant I of the *O174L* gene.

## Discussion

The ongoing pandemic spread of ASF threatens the global domestic pig industry with severe socio-economic consequences and this highlights the challenge posed by globalization in the rapid spread of infectious diseases. The knowledge of ASFV genomics provides important information required for an effective diagnosis and understanding of the disease transmission dynamics and control. Therefore, this study reports the first complete genome sequence of ASFV genotype XV. In addition, the first complete genome of ASFV genotype IX and three ASFV strains belonging to genotype II collected from Tanzania have been determined in this study using Illumina next-generation sequencing platform and comparative genomic analyses.

The Tanzanian ASFV genotype II strains reported in this study were closely related to genotype II ASFV isolates previously reported in Tanzania^29^, Malawi^35^, several European and Asian countries^8,36,37^ with more than 99.90% nucleotide identity. This high genetic identity between ASFV genotype II strains circulating worldwide underscores their likely common origin as previously reported^8,21^. For instance, the introduction of ASFV genotype II into Madagascar in 1998 most likely from Mozambique and subsequent spread to the island of Mauritius was suspected to be through swill feeding to domestic pigs^38,39^. Furthermore, genotype II of ASFV with high genetic similarity to isolates from Mozambique, Madagascar and Zambia was introduced into Georgia in 2007 through suspected feeding of infected pork product brought in by ship sailors to domestic pigs^8^. From Georgia, the virus spread to other European and Asian countries before it reached South America in 2021^10,40^. On the other hand, genotype II ASFV has been introduced in southern African countries where it was never known to circulate, for instance in Tanzania in 2010^27^ and Zimbabwe in 2015^41^. The re-emergence of ASF in Zimbabwe after a long period of absence was suspected to originate from neighbouring Mozambique^41^ while the introduction of the ASFV genotype II into Tanzania was suspected to be from Malawi^27^. Since the introduction of genotype II of ASFV in Tanzania, its persistent circulation in domestic pigs driven by uncontrolled domestic pig movements and low biosecurity has been reported^24^. In addition, a northward spread of genotype II ASFV in Tanzania has been reported threatening the domestic pig industry in the country with the risk of incursion in neighbouring countries of Rwanda, Burundi, Uganda, Kenya and the Democratic Republic of the Congo where this genotype is not known to circulate at the moment. In May 2020, ASFV genotype II was reported for the first time in Nigeria, a country in West Africa where only ASFV genotype I was prevalent^19^. Most of the genetic variations reported in this study were located at the left and right termini covering different members of MGFs including MGFs 110 and 360. These findings are in agreement with previous studies that have reported genetic variation at the 5′ and 3′ ends of the ASFV genomes belonging to genotype II^16,42,43^. The isolate TAN/17/Mbagala described in this study has a relatively short genome size compared to other ASFV genotype II isolates without a member of MGF 110 identified within its genome. The MGF 110 is classified among the most variable MGFs and considerable variations have been previously reported among members of MGF 110^44^. Most of copies of this MGF are located at the left variable region of the ASFV^45^. In our study, 17,641 bp at the 5′ end of the TAN/17/Mbagala ASFV isolate could not be sequenced.

For further discrimination among ASFV genotype II, the *K145R* and *O174L* genes were analyzed as previously described^32–34^. All ASFV genotype II isolates described in this study were 100% homologous to the reference genome Georgia 2007/1 classifying them into variant I of the *K145R* and *O174L* genes. Based on the *K145R* gene analysis, isolates characterized in this study showed 100% nucleotide identity at this particular marker to isolates previously described in European and Asian countries including in Belgium, China, Czech Republic and India. The *K145R* variant II isolates characterized by the presence of a single nucleotide polymorphism (SNP) within the *K145R* gene compared to the Georgia 2007/1 reference genome have been reported in Germany, Lithuania, Poland and Romania^34^. A unique cluster with two SNPs within the *K145R* gene has been described in isolates from Kaliningrad, Russia from 2017 to 2019^33^. The nucleotide identity between the isolates described in this study, and the *K145R* variant II and the Kaliningrad cluster was 99.77 and 99.54%, respectively. In addition, the ASFV genotype II isolates described in this study showed 100% nucleotide identity to Georgia 2007/1—like isolates at the *O174L* gene classifying them within variant I along with the majority of isolates from Europe and Asia^33,34^.

The complete genome sequence of the Tanzanian ASFV genotype IX described in this study showed 99.85% nucleotide identity with isolates responsible for ASF outbreaks in Uganda in 2015^46^. In Tanzania, ASFV genotype IX is restricted to the Lake Zone bordering Uganda and Kenya where this genotype is predominant^47,48^. The high genetic similarity between ASFV genotype IX circulating in Tanzania, Uganda and Kenya which share a common border highlights the possible transboundary transmission of this genotype between these countries as previously reported^22^. However, the Tanzanian Lake Zone where ASFV genotype IX is restricted is rich in wildlife protected areas with natural reservoirs of ASFV including warthogs and soft ticks which play a role in the epidemiology of ASFV sylvatic circulation. Besides, the ASFV genotype IX has been reported in the Democratic Republic of the Congo^20,49^ and in the Republic of the Congo^50^ underscoring the continuous spread of this genotype on the African continent with the risk of escaping from Africa to other continents that have a significant proportion of pork production at a global scale.

In this study, the first complete genome sequence of ASFV genotype XV has been described. At the moment, the ASFV genotype XV is restricted to Tanzania only and it has been reported in both domestic pigs and soft ticks^25,30,51^. This genotype is responsible for the 2001 and 2008 ASF outbreaks in Dar es Salam and Morogoro regions of Tanzania while the isolation from soft ticks was done in 2019 from Saadani National Park located in Tanga region^25,30,51^. The complete genome sequence of ASFV genotype XV described in this study showed 96.93% nucleotide identity with the Malawian ASFV isolate MalawiLil-20/1(1983). These findings are in agreement with those obtained using partial nucleotides amplification and sequencing where Tan/08/Mazimbu and MalawiLil-20/1(1983) isolates were found to be most closely related^30^ underscoring the overall good quality sequences generated in this study. Deletion partially covering *EP402R (CD2v)* and *EP153R* genes was detected in the ASFV genotype XV characterized in this study. Deletion covering the same genes has been reported in ASFV genotype II isolates leading to the loss of the hemadsorption abilities and reduction of virulence^16,42^. The isolate characterized in this study was collected from an intensive domestic pig farm in Mazimbu in Morogoro region of Tanzania and led to 97 deaths among 205 domestic pigs that were present at the farm at the time of the ASF outbreak^30^. The low mortality rate can be explained by reduced virulence of the ASFV strain as the farm was composed of Landrace and Large White crosses domestic pigs which are known to be highly susceptible to ASFV as compared to local domestic pig breed^52–54^. However, the effect of the genetic variations observed in this study on the phenotypic characteristics of the ASFV strain Tan/08/Mazimbu needs further investigation. The advantage of the approach used in this study is that ASFV complete genome sequencing was performed using DNA extracted directly from clinical samples minimizing the risk of potential genome modifications associated with in vitro culturing of the virus as previously reported^55,56^.

In conclusion, the complete genome sequences of ASFV genotypes II, IX and XV responsible for outbreaks in domestic pigs in Tanzania have been generated in this study. The ASFV genotype II described in this study mainly resembles the unique ASFV complete genome previously reported in Tanzania suggesting a possible common source and persistent circulation in the domestic pig population. In addition, the ASFV genotype IX generated in this study exhibited high genetic similarity with isolates previously reported in Tanzanian neighbouring countries underscoring the possibility of transboundary transmission. Interestingly, the first complete genome of ASFV genotype XV was generated in this study adding more value to further comparative genomic studies. The results of this study provide important insights into the genomic structure of ASFV strains circulating in Tanzania to monitor changes within the ASFV genome and improve our understanding of the ASF transmission dynamics for enhanced ASF risk management.

## Materials and methods

### Samples description

**Figure 5:**
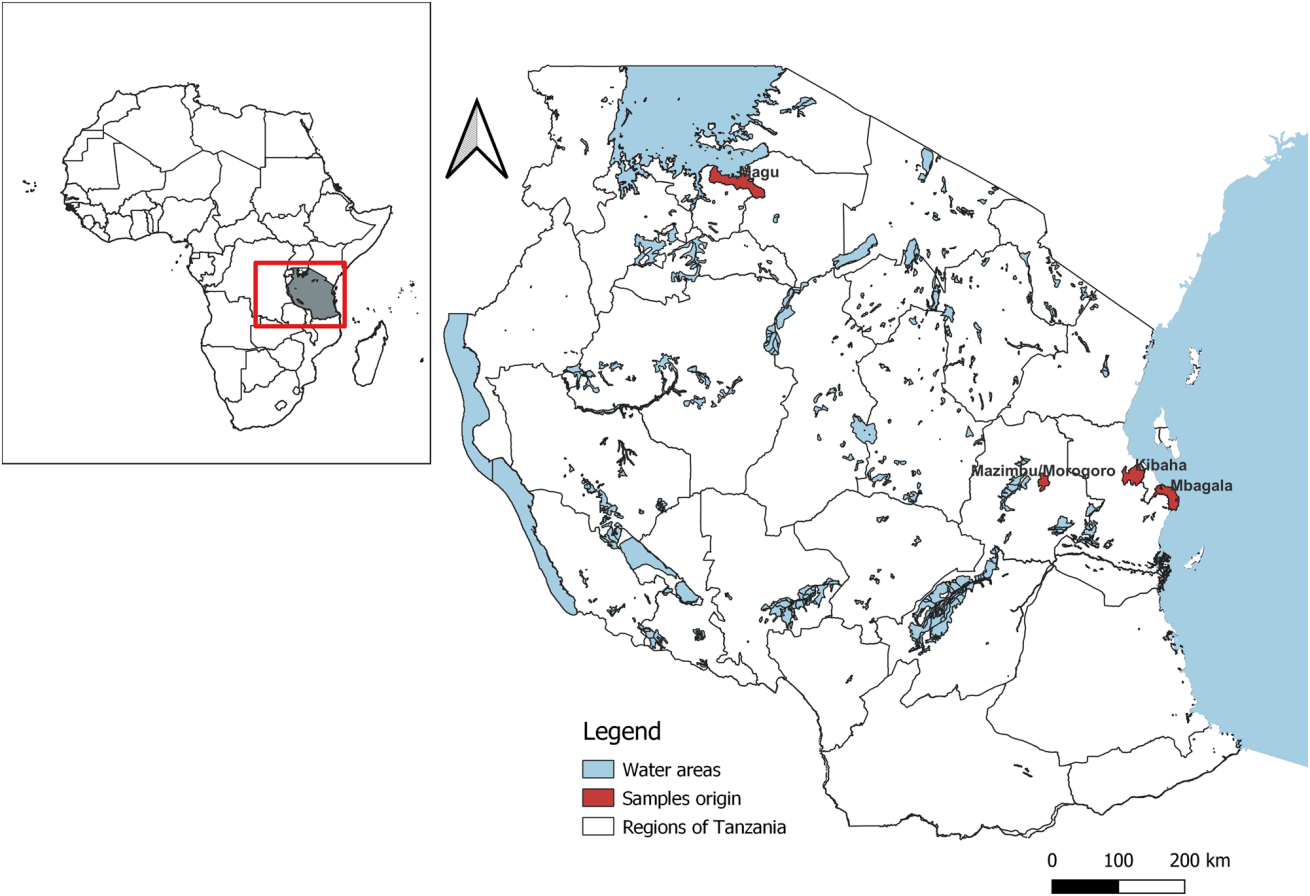
Map of Tanzania showing the origin of samples characterized in this study. Samples were collected from Magu district in Mwanza region, Mbagala in Temeke district of the Dar es Salaam region, Mazimbu in Morogoro urban district of Morogoro region, and from Kibaha district of the Coast region. The map was developed using QGIS software version 3.24.1 and data from DIVA-GIS freely available at https://www.qgis.org/en/site/ and https://www.diva-gis.org/Data, respectively.

Samples collection, processing, ASF confirmation and partial molecular characterization of the strains Tan/08/Mazimbu, TAN/17/Kibaha, TAN/17/Mbagala and TAN/16/Magu have been previously described and reported^24,26^. The strain TAN/20/Morogoro was collected during the 2020 ASF outbreak in domestic pigs in Morogoro region of Tanzania (Fig. 5). Tissue samples including spleen, hepatogastric lymph node and liver were collected from domestic pigs dead from a hemorrhagic disease with clinical signs suggestive of ASF. Samples were transported to the laboratory and the presence of ASFV was confirmed by polymerase chain reaction (PCR) by partial amplification of the *B646L* (p72) gene as previously described^57^. To classify the ASFV strains characterized in this study into the 24 ASFV genotypes already described to date, nucleotide amplification, sequencing and phylogenetic analysis of the C-terminal end of the *B646L* (p72) gene were conducted as previously reported^57^.

### DNA extraction

Genomic DNA was extracted from ASFV-positive samples using the Quick-DNA™ Miniprep Plus Kit (Zymo Research Corporation, CA, USA), following the manufacturer’s instructions. The purity and integrity of the extracted DNA were assessed using a nanodrop spectrophotometer (Biochrom, Cambridge, England) and 1% agarose gel electrophoresis, respectively. Picogreen method (Invitrogen, Catalog # P7589) using Victor 3 fluorometry (PerkinElmer Life and Analytical Sciences, Shelton, USA) was used to quantify the starting genomic DNA for library preparation.

### Library construction and quality assessment

According to the manufacturer’s protocol, the sequencing library preparation was performed using TruSeq Nano DNA Kit (Catalog # 20,015,964; Illumina, USA). The prepared libraries were subjected to quality control using 2100 Bioanalyzer with a DNA 1000 chip (Agilent Technologies, USA) and quantification using real-time polymerase chain reaction (qPCR) according to the Illumina qPCR Quantification Protocol Guide (Catalog # SY-930-1010).

### Complete genome sequencing of the Tanzanian ASFV strain*s*

The genomic DNA libraries were subjected to paired-ends sequencing using Illumina NovaSeq6000 instrument with 2 × 150 bp configuration and approximately 28 million paired-end reads (4 gigabytes) per sample were produced. To exclude the gap through the *EP402R (CD2v)* gene of ASFV strain Tan/08/Mazimbu, this genomic region was partially amplified and subjected to Sanger sequencing using the following primers pair: EP402R middle forward (5′–TATCAGTATAATACACCTATTTACTA–3′) and EP1 reverse (5′–ACATGATGTTCTCGATGATC–3′).

### Bioinformatics analysis

After sequencing, the quality control of the raw sequencing reads was performed using FastQC version 0.11.9^58^ to get information on the overall quality of the generated reads, total bases, total reads, GC content and basic statistics. To avoid biases in the analysis, sequencing adapter trimming and quality filtering were performed and the quality of the filtered reads was assessed again. Trimming of sequencing adapters and filtering of low-quality sequencing reads were performed using Trim Galore version 0.6.4 with cutadapt version 2.8 with the quality Phred score cutoff set to 30 with a minimum read length of 75 base pairs. The filtered reads were mapped against ASFV reference genomes Georgia2007/1 (GenBank accession number FR682468.2) and Ken06.bus (GenBank accession number NC_044946.1) using Burrows-Wheeler Aligner (BWA) version 0.7.17 with a maximum exact match (mem) option^59^. The sequencing reads of the strain Tan/08/Mazimbu were mapped to the most closely related ASFV isolate at the NCBI GenBank which was MalawiLil-20/1(1983) (GenBank accession number AY261361). Using the mapped reads, de novo assembly was performed using SPAdes version 3.13.1^60^ and the quality of the assembly was assessed using QUAST program version 5.0.2^61^. The longest contig for each sample was considered as the ASFV complete genome. After complete genome assembly, the location of protein-coding sequences was identified and annotated using the Genome Annotation Transfer Utility (GATU) software^62^ with Georgia2007/1 and Ken06.bus as reference genomes. For phylogenetic analysis, multiple sequence alignment of complete genome sequences of ASFV strains characterized in this study together with isolates previously reported from NCBI GenBank was performed using the MAFFT program version 7.221^63^ and the most suitable model for phylogeny reconstruction was selected using smart model selection in PhyML (SMS) version 1.8.4^64^ via Akaike information criterion (AIC). The maximum likelihood phylogenetic tree was reconstructed using the general time reversible model and rates among sites set to gamma distributed with invariant sites (GTR + G + I) as well as 1000 bootstrap replications as implemented in MEGA X^65^.

### Ethics statement

No ethical approval was required for this study as the samples used were collected from naturally dead domestic pigs according to common veterinary practice as part of a routine veterinary investigation in Tanzania according to the Animal Diseases Act, 2003 (No. 17 of 2003).

## Supplementary Information

Supplementary Table 1.

## Data Availability

The complete nucleotide sequences generated in this study are available at NCBI GenBank under Accession Numbers ON409979 to ON409983.
